# Efficacy and Safety of Tranexamic Acid in Aneurysmal Subarachnoid Hemorrhage: A Systematic Review and Meta-Analysis of Randomized Controlled Trials

**DOI:** 10.3389/fsurg.2021.790149

**Published:** 2022-01-10

**Authors:** Min Shi, Chao Yang, Zu-han Chen, Ling-fei Xiao, Wen-yuan Zhao

**Affiliations:** ^1^Department of Neurosurgery, Zhongnan Hospital of Wuhan University, Wuhan, China; ^2^Institute of Hepatobiliary Diseases of Wuhan University, Zhongnan Hospital of Wuhan University, Wuhan, China; ^3^Department of Orthopaedics, Zhongnan Hospital of Wuhan University, Wuhan, China

**Keywords:** tranexamic acid, aneurysmal subarachnoid hemorrhage, mortality, poor outcome, rebleeding, adverse events

## Abstract

Tranexamic acid has been shown to reduce rebleeding after aneurysmal subarachnoid hemorrhage; however, whether it can reduce mortality and improve clinical outcomes is controversial. We performed a systematic review and meta-analysis to evaluate the efficacy and safety of the tranexamic acid in aneurysmal subarachnoid hemorrhage. We conducted a comprehensive literature search of PubMed, Embase, Web of Science, and Cochrane Library from inception to March 2021 for randomized controlled trials (RCTs) comparing tranexamic acid and placebo in adults with aneurysmal subarachnoid hemorrhage. The risk of bias was evaluated using the Cochrane Handbook, and the quality of evidence was evaluated using the GRADE approach. This meta-analysis included 13 RCTs, involving 2,888 patients. In patients with aneurysmal subarachnoid hemorrhage tranexamic acid had no significant effect on all-cause mortality (RR = 0.96; 95% CI = 0.84–1.10, *p* = 0.55, *I*^2^ = 44%) or poor functional outcome (RR = 1.04; 95% CI = 0.95–1.15, *p* = 0.41) compared with the control group. However, risk of rebleeding was significantly lower (RR = 0.59; 95% CI = 0.43–0.80, *p* = 0.0007, *I*^2^ = 53%). There were no significant differences in other adverse events between tranexamic acid and control treatments, including cerebral ischemia (RR = 1.17; 95% CI = 0.95–1.46, *p* = 0.15, *I*^2^ = 53%). At present, routine use of tranexamic acid after subarachnoid hemorrhage cannot be recommended. For a patient with subarachnoid hemorrhage, it is essential to obliterate the aneurysm as early as possible. Additional higher-quality studies are needed to further assess the effect of tranexamic acid on patients with subarachnoid hemorrhage.

## Introduction

Aneurysmal subarachnoid hemorrhage (aSAH), referring to the release of arterial blood into the subarachnoid space due to the ruptured of an intracranial aneurysm, is a common, devastating emergency cerebrovascular disease worldwide (1, 2). aSAH is a serious type of stroke, accounting for approximately 5% of all stroke (3). In spite of progress and development in the diagnosis and treatment of aSAH, the mortality rates are still high (4). Increasing studies indicated that rebleeding is a leading cause of high mortality and poor outcome in the patients of aSAH (5). Rebleeding rate are up to 20% from ruptured aneurysms, and its highest incidence is observed in the first 24 h after the initial hemorrhage (6–9). The current evidence suggests that effective aneurysm treatment as early as possible is the best way to reduce or even to prevent rebleeding after hemorrhage (10–12). However, not all patients are treated immediately within a few hours after admission (13, 14). Therefore, a more effective treatment modality, not just aneurysm surgery alone, is still needed before the aneurysm is secured.

Tranexamic acid (TXA) is a common anti-fibrinolytic agent that act by inhibiting the activity of plasminogen to plasmin (15, 16). TXA has been widely used to reduce blood loss from surgery, severe traumatic injury and heavy menstruation in recent years (17–20). In 1967, antifibrinolytic therapy used for SAH first reported by Gibbs in 1967 (21). Afterward, numerous studies have explored the effect of TXA in aSAH. However, whether TXA is effective in SAH has been controversial (22–25). Although most studies have demonstrated that TXA can reduce rebleeding, they failed to show that it improves poor outcomes and mortality (26, 27). A previous meta-analysis published in 2013 showed that antifibrinolytic therapy can reduce risk of rebleeding but cannot improve clinical outcome or reduce mortality (25). It is likely that an increase in the risk of cerebral ischemia offset the effect of TXA on rebleeding (24, 25). However, a recent study demonstrated that TXA may have a significant benefit on mortality in non-traumatic intracranial bleeding patients (28). Given the conflicting evidence, we cannot conclude whether TXA should routinely be used in patients with aSAH. Therefore, there is still a need to reevaluate the efficacy and safety of TXA in aSAH.

## Materials and Methods

### Search Strategy

A meta-analysis was performed using methods proposed in the Preferred Reporting Items for Systematic Reviews and Meta-Analyses (PRISMA) guidelines (Supplementary File 1). We conducted a comprehensive literature search from inception to March 2021 in the following databases: PubMed, EMBASE, the Cochrane Library, and Web of Science. The search strategies were established with search terms as follows: (subarachnoid hemorrhage OR SAH) AND (tranexamic acid OR TXA). The detailed search terms are listed in Supplementary File 2. In addition, we also manually searched reviews and the references therein to prevent the omission of eligible literature.

### Selection Criteria

The studies available for the meta-analysis met the following inclusion criteria: (1) RCT study design; (2) inclusion of study participants diagnosed with SAH due to suspected or confirmed ruptured aneurysm; (3) intervention treatment with TXA vs. matched placebo treatment; and (4) inclusion of at least one of relevant outcome measures.

The exclusion criteria were as follows: (1) study designs other than RCT; (2) study participants not diagnosed with aSAH; (3) intervention treatments were not TXA vs. matched placebo treatment; (4) none of the relevant outcome measures included; and (5) insufficient data or excessive missing data.

### Outcome Measures

Efficacy outcome measures: The primary efficacy outcome used to assess the efficacy of TXA in aSAH was all-cause mortality at the end of the follow-up. The secondary efficacy outcomes included poor outcome and risk of rebleeding.

All-cause mortality was defined as death from any cause at the final follow-up, and the causes included rebleeding, cerebral ischemia, hydrocephalus, other extracranial causes, and complications of operation or anesthesia. Poor outcomes were assessed with the modified Rankin Scale (mRS≥4) or Glasgow Outcome Scale (GOS ≤ 3) at the final follow-up. Rebleeding included definite [confirmed by computed tomography (CT) scan, or at necropsy, or in the cerebrospinal fluid (CSF)] or possible (sudden neurological deterioration with change in vital parameters suggestive of recurrent bleeding not confirmed by CT, or sudden increase of fresh blood production from an external ventricular drain).

Safety endpoints: The safety of the TXA was evaluated by examining the occurrence of serious adverse events. The main adverse events in the meta-analysis included hydrocephalus, cerebral ischemia, and venous thromboembolism (VTE).

### Study Selection and Data Extraction

Two researchers independently screened each article in accordance with the predefined inclusion and exclusion criteria. When a disagreement existed between the two researches, they attempted to achieve a consensus through discussion. The researchers independently extracted available data using a predefined checklist. The extracted data were as follows: first author, publication year, country, total number of patients, intervention and placebo details, follow-up time, and relevant outcome data.

### Quality Assessment

We assessed the risk of bias of each study using the methods provided in the Cochrane Handbook for Systematic Reviews of Interventions. The two researches independently conducted the assessment according to the seven aspects: random sequence generation; allocation concealment; blinding of participants and personnel; blinding of outcome assessment; incomplete outcome data; selective outcome reporting; and other bias; each of them was classified into high, low, or unclear based on the information provided by each study. When a disagreement existed between the two researches, they attempted to resolve the disagreement through discussion.

We assessed the quality of evidence for each outcome using the Grading of Recommendations, Assessment, Development, and Evaluation (GRADE) approach. The GRADE guideline classifies the quality of evidence as high, moderate, low, or very low.

### Statistical Analysis

We conducted the meta-analysis by using RevMan 5.4 software (Review Manager, Cochrane Library, Oxford, UK, The Cochrane Collaboration, 2020). The results were represented by the forest plots. Pooled odds ratios (ORs) with 95% confidence intervals (CIs) were used as summary statistics and were calculated from the comparison of tranexamic acid with placebo. Heterogeneity was defined as *I*^2^ > 50% or *P* < 0.10. We adopted a fixed-effect random to synthesize data if heterogeneity was not significant; otherwise, a random effects model was applied. When the results demonstrated significant heterogeneity, we performed subgroup and sensitivity analyses to examine the potential source of heterogeneity.

## Results

### Literature Selection and Characteristics of the Included Studies

**Figure 1:**
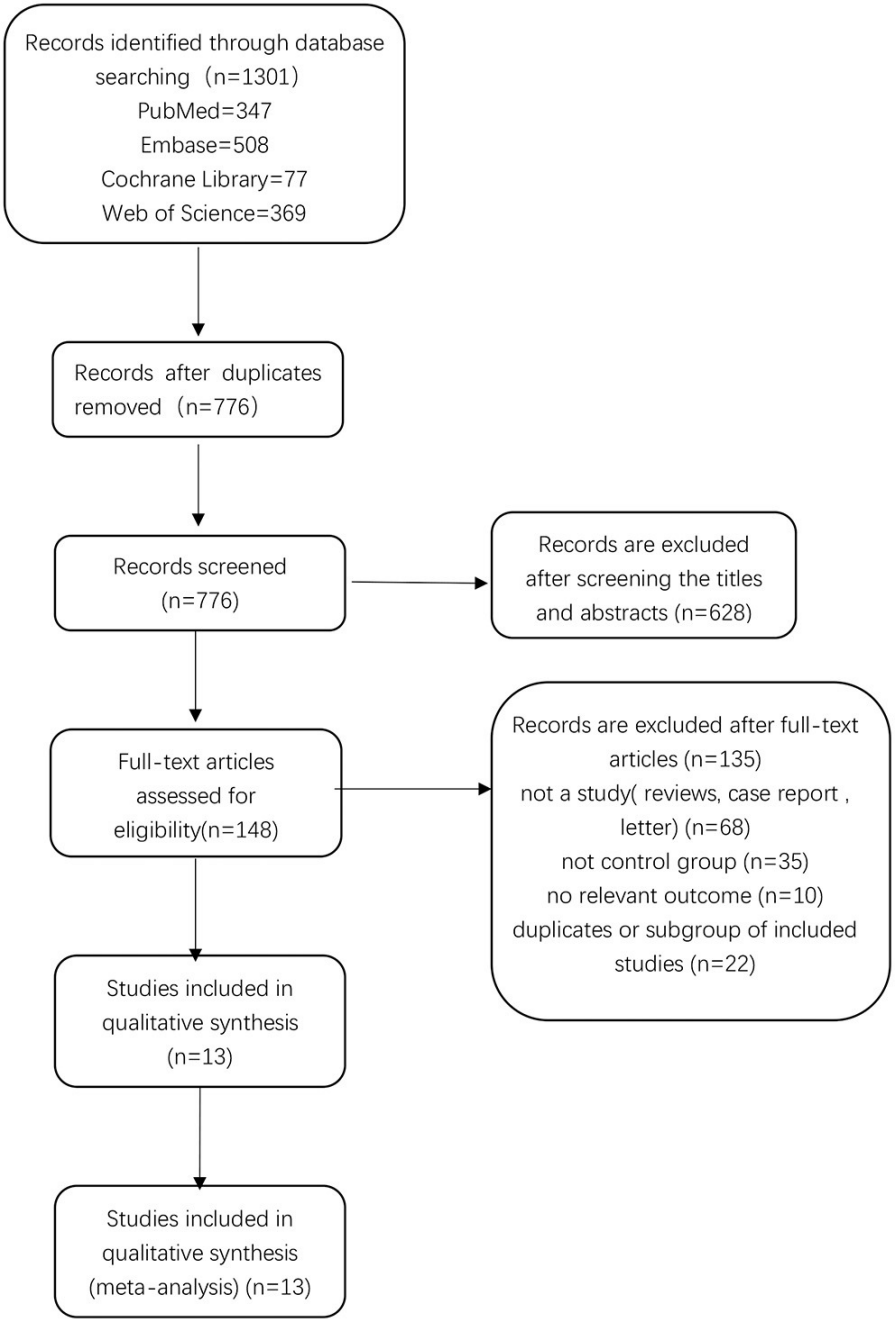
Flow chart of the study selection process.

There were 1,301 relevant records identified in the initial database search. After excluding 525 duplicates, 776 records remained. By screening titles and abstracts, we excluded 628 records, leaving 148 records for which we read the full-text articles to sort out the eligible studies. Eventually, we included 13 studies in the final analysis (26, 27, 29–39). Two of these studies were published by the same author (Harald Fodstad) in 1981 (32, 33), but the data were not duplicate. A flow chart showing the selection process is presented in Figure 1.

The characteristics of the included trials are provided in Table 1. Ultimately, we selected 13 studies, including 2,888 patients in the meta-analysis. A total of 1,451 patients were treated with tranexamic acid, while 1,437 received control treatment, including standard treatment or placebo treatment. The sample size of each study ranged from 39 to 505. All but one of the included studies were conducted in Europe. Five RCTs were multicenter studies, while eight were conducted at a single site. Most of the studies were published before 2,000, and only three studies were published after 2,000. Clinical status at admission were evaluated and graded according to Hunt-Hess scale, WFNS grade, Glasgow Coma Scale or Botterell scores. The duration of tranexamic acid treatment differed considerably between the studies and ranged from <72 h up to 6 weeks. In the latest two studies, short-term treatment (<72 h) was used. In three studies, participants were concomitantly treated with measures to prevent or reverse cerebral ischemia. The follow-up period ranged from 3 weeks to 43 months. TXA was administrated intravenously in all studies except one in which it was administered orally. The dosage and usage of TXA was similar among the included trials, with the most common administration being a first dose of 1 g, followed by a different maintenance dose.

**Table 1:** Baseline characteristics of the included studies.

**Study**	**Country**	**Design**	**Patients**		**Mean age**		**Clinical Scores**	**FUP**	**TP**	**Protocol**
			**TT**	**PT**	**TT**	**PT**				
Post (29)	Netherlands	multicenter RCT	480	474	58.4 (12.6)	58.4 (12.3)	WFNS	6 m	24 h	IV 1 g, directly followed by 1 g/8 h
Hillman (27)	Sweden	multicenter RCT	254	251	>15	>15	Fisher/Hunt-Hess	6 m	3 d	IV 1 g bolus then 1 g 2 h after bolus then 1 g/6 h
Roos (26)	Netherlands	multicenter RCT	229	233	55 (14.0)	56 (14.0)	GCS	3 m	3 w	IV 6 g/d bolus 1 w, then 6 g/d po 2 w
Tsementzis (30)	England	single center RCT	50	50	>20	>20	Botterell	6 m	4 w	IV 1.5 g/4 h 4 w
Vermeulen (31)	Netherlands	multicenter RCT	241	238	50.3	50.2	Hunt-Hess	3 m	4 w	IV 6 g/d bolus 2 w, IV or orally 6 g/d 2 w
Fodstad (32)	Sweden	single center RCT	30	29	50 (19–72)	53 (27–70)	Botterell	41 m	6 w	IV 1 g/4 h 1 w, then IV 1 g/6 h 1 w, then 1,5 g/6 h orally 4 w
Fodstad (32)	Sweden	single center RCT	21	20	49 (25–64)	45 (23–70)	Botterell	5 w	5 w	IV 1 g/4 h 1 w, then 1 g/6 h 4 w
Kaste (34)	England	single center RCT	32	32	<61	<61	Botterell	3 w	3 w	IV 1 g/4 h
Maurice (37)	England	single center RCT	25	25	<65	<65	Botterell	33 m	6 w	IV 1 g/4 h 1 w, then 1.5 g/6 h per mouth
Fodstad (35)	Sweden	single center RCT	23	23	45 (23–68)^a^ 51 (29–66)^b^	45 (23–68)^a^ 51 (29–66)^b^	Botterell	34 m	6 w	IV 1 g/4 h 1 w, then 1 g/6 h 4 w then 1 g/8 h 1 w
Chandra (36)	Indonesia	single center RCT	20	19	51 (20–65)	51 (20–65)	Botterell	3 w	3 w	IV 1 g/4 h 1 w then 1 g/6 h 3 w
Rossum (38)	Netherlands	multicenter RCT	26	25	54	58	NR	3 m	10 d	IV 1 g/6 h
Gibbs (39)	England	single center RCT	22	25	49.9	56.5	NR	2 m	3 w	3 g/day orally

*TT, TXA treatment; PT, placebo treatment; FUP, follow-up period; TP, treatment period; RCT, randomized controlled trial; m, month; w, week; d, day; h, hour; GCS, Glasgow coma scale; NR, not reported; a, age for men; b, age for women*.

### Efficacy Outcomes of Tranexamic Acid

#### All-Cause Mortality at the Final Follow-Up

**Figure 2:**
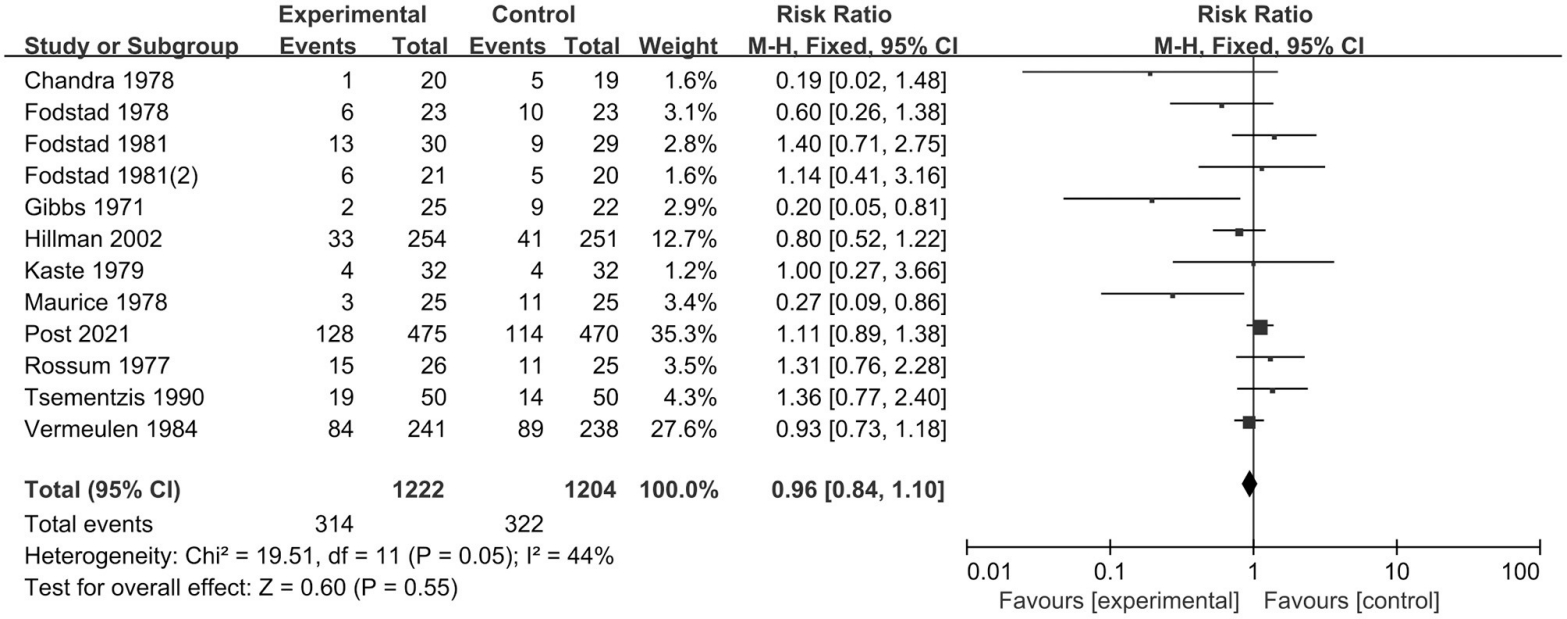
Forest plot comparing tranexamic acid and control treatment for the outcome of all-cause mortality.

A total of 12 studies including 2,426 patients analyzed the occurrence of all-cause mortality. We selected a fixed-effect model due to significant heterogeneity (*I*^2^ = 44%; *p* = 0.05). The pooled result indicated that TXA had no significant effect on mortality compared with the control treatment (RR = 0.96; 95% CI = 0.84–1.10, *p* = 0.55) (Figure 2). The subgroup analysis stratified by treatment duration indicated that the short-term TXA treatment (≤3 days) had no significant effect on mortality compared with the control treatment (RR = 1.03; 95% CI = 0.85–1.25, *p* = 0.78), and long-term TXA treatment (> 3 days) had no significant effect on mortality compared with the control treatment (RR = 0.90; 95% CI = 0.75–1.07, *p* = 0.24) (Supplementary File 3). The subgroup analysis stratified by follow-up period indicated that TXA treatment had no significant effect on short-term (≤ 90 days) mortality compared with the control treatment (RR = 0.95; 95% CI = 0.81–1.11, *p* = 0.50, *I*^2^ = 29%); likewise, TXA treatment had no significant effect on long-term (>90 days) mortality compared with the control treatment (RR = 0.99; 95% CI = 0.83–1.17, p = 0.49, *I*^2^ = 58%) (Supplementary File 4).

#### Poor Functional Outcome

**Figure 3:**
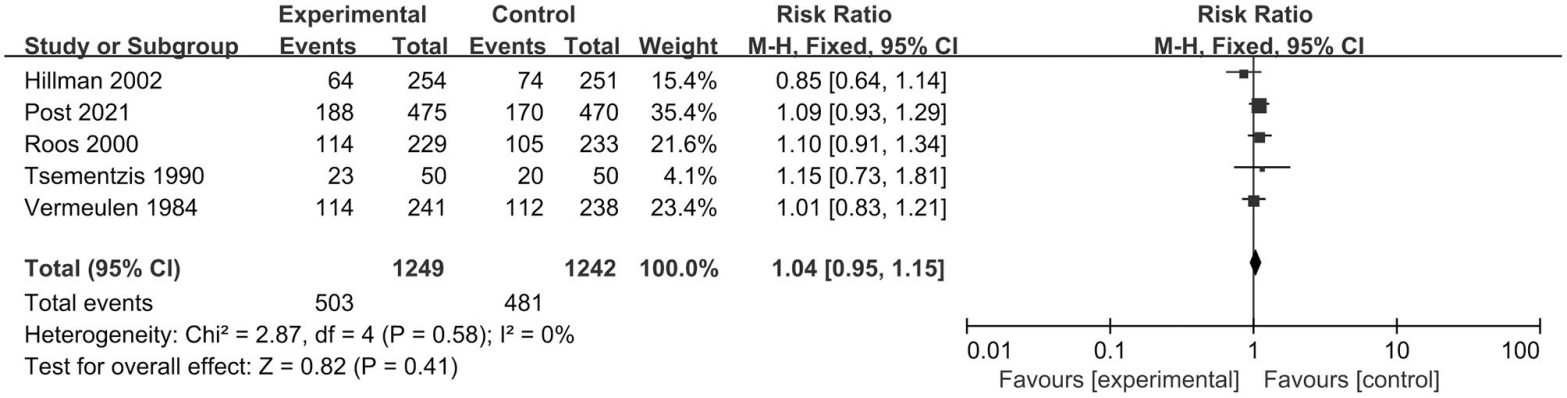
Forest plot comparing tranexamic acid and control treatment for the outcome of poor functional outcome.

In total, five RCTs, including 2,491 patients, analyzed poor clinical outcomes. We selected a fixed-effect model due to significant heterogeneity (*I*^2^ = 0%; *p* = 0.58). The pooled result indicated that tranexamic acid had no significant effect on the poor functional outcomes compared with the control treatment (RR = 1.04; 95% CI = 0.95–1.15, *p* = 0.41) (Figure 3).

#### Rebleeding

**Figure 4:**
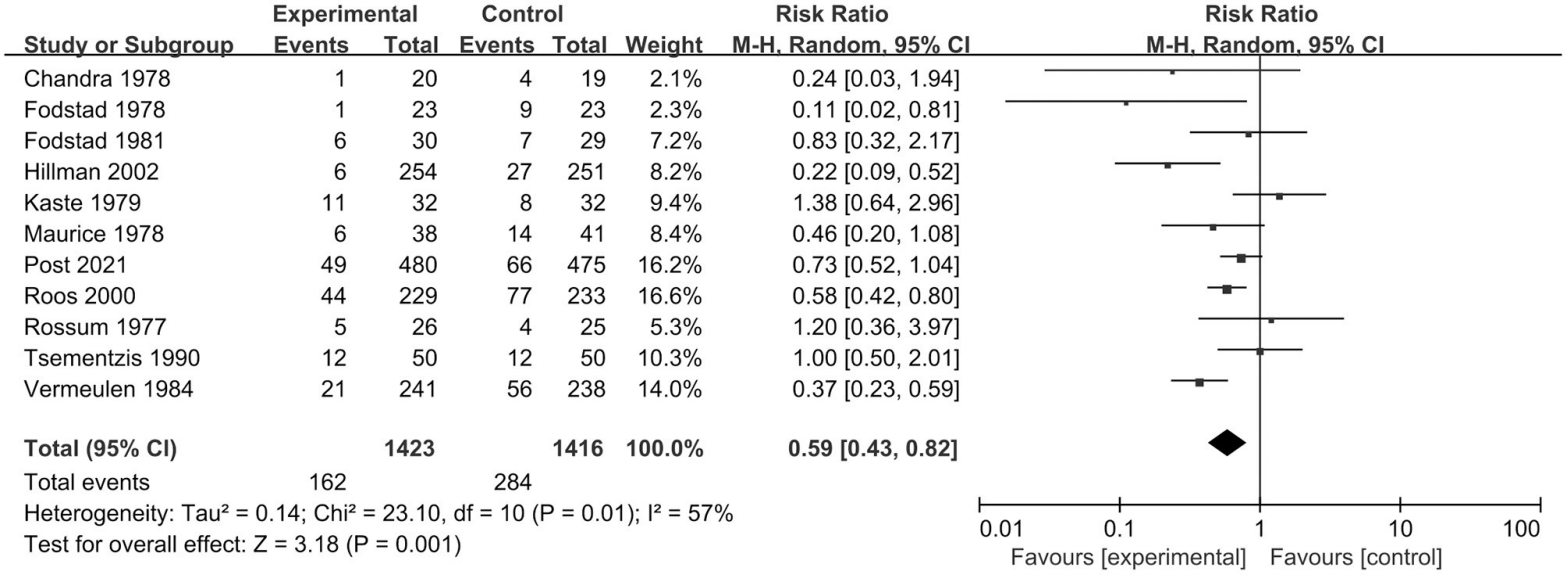
Forest plot comparing tranexamic acid and control treatment for the outcome of rebleeding.

In total, 12 RCT studies, including 2,851 patients, compared the occurrence of rebleeding in the tranexamic acid and control treatment. We selected a random-effect model for the meta-analysis due to significant heterogeneity (*I*^2^ = 53%; *p* = 0.02). The pooled results indicated that tranexamic acid had significant effect on rebleeding compared with the control treatment (RR = 0.59; 95% CI = 0.43–0.80, *p* = 0.0007) (Figure 4). Due to the study heterogeneity, we conducted a sensitivity analysis by removing each study in turn. The heterogeneity did not obviously decrease after excluding any study, and the results of sensitivity analysis were consistent with the overall results. Overall, sensitivity analyses showed that the results were stable.

#### Safety Outcomes of Tranexamic Acid

The occurrence of the most common adverse events between tranexamic acid and placebo was not significantly different (Supplementary File 5). In adverse events, cerebral ischemia was the most common and serious side-effect that was the second most common cause of death. Eight studies, including 2,646 patients, reported the occurrence of cerebral ischemia or cerebral infarction. The pooled result indicated no statistically significant difference in cerebral ischemia between TXA treatment and control treatment (RR = 1.17; 95% CI = 0.95–1.46, *p* = 0.15, *I*^2^=53%). Seven studies, including 2,180 patients, reported the occurrence of hydrocephalus. The pooled result indicated no statistically significant difference between TXA treatment and control treatment (RR = 1.09; 95% CI = 0.99–1.20, *p* = 0.10, *I*^2^ = 6%). Seven studies, including 2,151 patients, reported the occurrence of DVT. The pooled result indicated no statistically significant difference between TXA treatment and control treatment (RR = 1.16; 95% CI = 0.75–1.80, *p* = 0.50, *I*^2^= 0%).

#### Quality Assessment and GRADE Assessment of the Included Studies

The risk of bias of the included studies was assessed using the guidance published in the Cochrane Handbook for Systematic Reviews of Interventions. We assessed the risk of bias according to the following domains: random sequence generation; allocation concealment; blinding of participants and personnel; blinding of outcome assessment; incomplete outcome data; selective outcome reporting; and other bias (Supplementary File 6).

We used the GRADE approach to evaluate the quality of evidence of primary outcomes, classifying the quality of evidence as high, moderate, low, or very low (Table 2).

**Table 2:** GRADE summary of quality of evidence for primary outcomes: tranexamic acid vs. control treatment in aneurysmal subarachnoid hemorrhage.

**Quality assessment**							**No of patients**		**Effect**		**Quality**	**Importance**
**No of studies**	**Design**	**Risk of bias**	**In consis tency**	**In direct ness**	**Impre cision**	**Other conside rations**	**TXA**	**Control**	**Relative (95% CI)**	**Absolute**		
All-cause mortality												
12	RCTs	Serious^b^	not serious	not serious	Serious^c^	none	417/1702 (24.5%)	426/1679 (25.4%)	RR 0.97 (0.86 to 1.08)	8 fewer per 1,000 (from 36 fewer to 20 more)	⊕⊕○○ LOW	CRITICAL
Poor outcome												
5	RCTs	serious^b^	not serious	not serious	Serious^c^	none	503/1249 (40.3%)	481/1242 (38.7%)	RR 1.04 (0.95 to 1.15)	15 more per 1,000 (from 19 fewer to 58 more)	⊕⊕○○ LOW	CRITICAL
Rebleeding												
11	RCTs	serious	Serious^a^	not serious	not serious	none	162/1423 (11.4%)	284/1416 (20.1%)	RR 0.59 (0.43 to 0.82)	82 fewer per 1,000 (from 36 fewer to 114 fewer)	⊕⊕○○ LOW	CRITICAL
Cerebral ischemia												
8	RCTs	Serious^b^	Serious^a^	not serious	Serious^c^	none	384/1328 (28.9%)	340/1318 (25.8%)	RR 1.17 (0.95 to 1.46)	44 more per 1,000 (from 13 fewer to 119 more)	⊕○○○ VERY LOW	IMPORTANT
Hydrocephalus												
7	RCTs	Serious^b^	not serious	not serious	Serious^c^	none	436/1091 (40%)	399/1089 (36.6%)	RR 1.09 (0.99 to 1.2)	33 more per 1,000 (from 4 fewer to 73 more)	⊕⊕○○ LOW	IMPORTANT
VTE												
7	RCTs	Serious^b^	not serious	not serious	Serious^c^	none	40/1078 (3.7%)	34/1073 (3.2%)	RR 1.16 (0.75 to 1.8)	5 more per 1000 (from 8 fewer to 25 more)	⊕⊕○○ LOW	IMPORTANT

a *Serious imprecision due to high I^2^ (>50%) and non-overlapping confidence intervals*.

b *Serious risk of bias due to lack of blinding of participants and personnel in some studies*.

c *Serious imprecision due to confidence interval including benefit and harm or low number of events below optimal information size*.

#### Publication Bias

**Figure 5:**
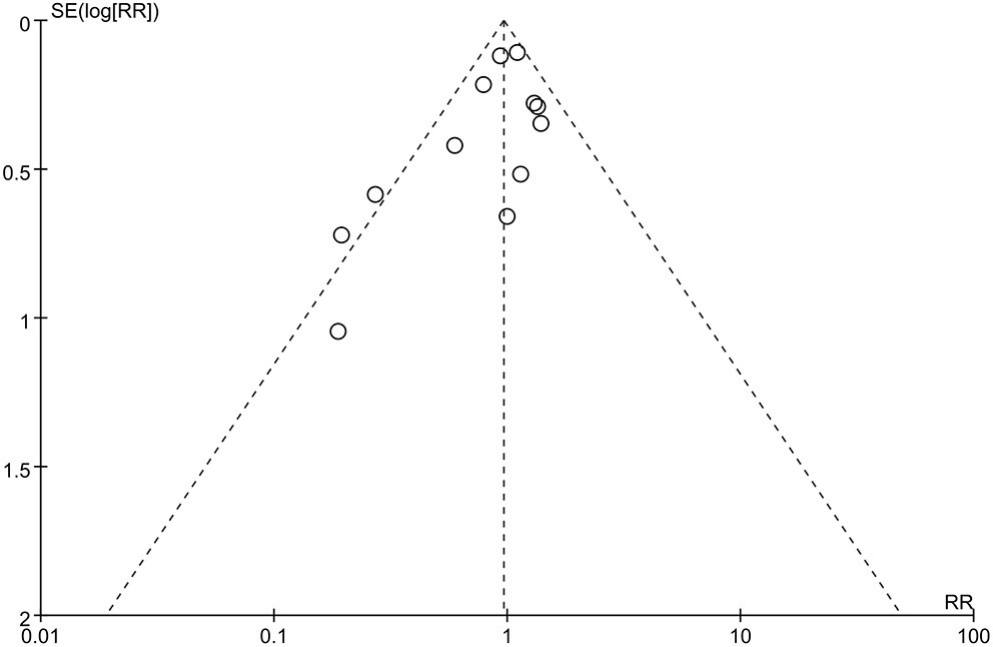
Funnel plot of publication bias of all-cause mortality: no significant publication bias was found.

To assess the possible publication bias, we performed funnel plots for the primary outcome (mortality). From the funnel plots of the primary outcome, we found no obvious publication bias (Figure 5).

## Discussion

In the systematic review and meta-analysis, we focused on assessing the efficacy and safety of TXA on aSAH. We included a total of 13 studies consisting of 2,888 patients. The results of our meta-analysis indicated that TXA may reduce the risk of rebleeding. However, we did not observe a beneficial effect on mortality or outcome. Likewise, TXA may not increase the occurrence of adverse events, including cerebral ischemia.

aSAH is one of the most common and serious neurosurgical conditions, with high morbidity and mortality rates (40, 41). Numerous studies have explored innovative therapeutic strategies to improve poor prognosis. In a previous meta-analysis of 10 RCTs consisting of nine TXA trials and one EACA trial (25), antifibrinolytic therapy after SAH was shown to reduce the risk of rebleeding and increase the occurrence of cerebral ischemia; however, there was no evidence of a beneficial effect on mortality and outcome. With the publications of ULTRA trial (29), there is a need to reevaluate the effect of TXA in aSAH patients.

The findings of the current study are comparable with previous systematic reviews and meta-analyses regarding the use of TXA in aSAH (24, 25, 28). In 2013, Baharoglu et al. evaluated the effect of antifibrinolytic therapy in aSAH; the study included two antifibrinolytic drugs, TXA and epsilon-aminocaproic acid (25). The authors found that antifibrinolytic therapy reduced the risk of rebleeding, but increased the risk of cerebral ischemia. However, our study indicated that TXA did not increase the risk of cerebral ischemia. Although neither of the two studies observed the reduction in mortality, the results of our meta-analysis showed a tendency toward reducing mortality unlike those of Baharoglu et al. (25). Regarding the included studies, Baharoglu et al. (25) had not included the studies by Post (29), Gibbs (39), Fodstad (35), and Fodstad (32), which were included in our study. In 2021, Bouillon-Minois et al. investigated the mortality in patients receiving TXA for nontraumatic intracranial bleeding, comprising SAH and intracranial bleeding, not aSAH alone (28). The results of their subgroup analysis showed a reduction in mortality in SAH, which was not in agreement with our study. In addition, we not only evaluated the effect of TXA on mortality, but also the effect of TXA on poor outcome, rebleeding, or adverse events, mainly including cerebral ischemia, hydrocephalus, or VTE.

Although the efficacy of TXA may be limited, our results showed that the use of TXA did not increase the occurrence of adverse events, including cerebral ischemia. Our finding is consistent with the recent large-scale RCTs (27, 29). Rebleeding is the most threatening early complication and predictor of poor outcome after aSAH (12, 42). The current study and previous studies demonstrated that TXA can effectively reduce the rebleeding rate, albeit without improving patient outcomes. In the previous meta-analysis, Baharoglu et al. have suggested that a significantly higher incidence of cerebral ischemia can offset the potentially positive effect of the reduced risk of rebleeding, which would lead to the lack of the beneficial impact on mortality or outcome (25). Recent studies have suggested that TXA treatment with short-term duration or with ischemia prevention can reduce the risk of rebleeding, without increasing the risk of cerebral ischemia (27). Our findings are consistent with those. However, we did not observe any significant improvement in patient clinical outcomes in the case of absence of cerebral ischemia. Hillman et al. (27) suggested that TXA effectively reduced the mortality and morbidity caused by very early rebleeding episodes although it did not improve the overall outcomes (27). Post et al. (29) have suggested that early brain injury or not-predefined complications may contribute to poor clinical outcome (29).

For patients with aSAH, long-term clinical prognosis is most concerning (29, 43). The current guideline calls for ultra-early repair of the aneurysm within first hours (44, 45). However, many patients after SAH are unable to reach the hospital timely for an aneurysm surgery. Park et al. (46) has reported a higher incidence of rebleeding in individuals who received TXA treatment within 72 hours compared with more recent patients who received TXA treatment immediately after admission; the prevention of rebleeding led to much better outcomes for the immediate-treatment group (46). In recent studies, short-term TXA treatment with ischemia prevention has been shown to reduce the mortality rate and increase the rate of favorable overall management outcome (27, 29). If we focus on reducing other complications during the disease course after the usage of TXA, patients' outcomes will likely be more positive.

There is no consensus how to use tranexamic acid for aSAH and whether the use of TXA is appropriate in this context. Some clinicians may support the use of TXA due to the reduction of rebleeding, absence of demonstrable harm, and potential improvement of clinical outcomes, while others advocate not using TXA due to the lack of demonstrable benefit (26, 27, 29, 31). TXA probably has a positive effect in certain subgroups, such as those stratified by the severity of hemorrhage ictus, location of hemorrhage, or different comorbidities. Our findings will probably be helpful for further studies addressing the topic of aSAH. In the future, we need more randomized controlled studies with large series to clarify the existence of a positive effect of TXA in specific subgroups.

In the future, we need to conduct more studies to improve the rebleeding rate and poor prognosis of such patients. First, with the most rebleeding occurring within 3 h after the initial hemorrhage, it is impossible to prevent all episodes of rebleeding by surgery. Subsequently, we recommend that further treatment allows the first medical staff who have initial contact with the patient to administer the TXA for short-term treatment in the future, which may buy time for emergency surgery. Second, the concept of “time is brain” has been well established. Once aSAH is diagnosed, we need to reduce the time from discovery to treatment to ensure treatment as soon as possible. Third, advances and strengthening in critical care management of patients with ruptured aneurysms, such as blood pressure control, may reduce the rate of rebleeding and improve long-term clinical outcome. Finally, patients with subarachnoid hemorrhage may develop many related serious complications leading to poor prognosis during the course of the disease. Therefore, TXA treatment for long time at the late stage of hemorrhage should be avoided. In the future, our treatment trend should focus on reducing the complications to improve outcomes and mortality.

There are some strengths of this study. We comprehensively searched all eligible RCTs, independently screened the included studies, extracted the data, assessed the quality of the included studies, and used the GRADE approach to evaluate the quality of evidence. In addition, we included newest trials to increase the overall sample size, thereby increasing the statistical power of our study. There are also some limitations. First, there was some clinical heterogeneity among the included trials, including the severity of hemorrhage, the hematoma volume, and the severity of comorbidities. Second, there were some methodological differences. Some studies lacked the blinding and the allocation concealment. Third, the administration regimen of TXA was not uniform among the studies.

## Conclusion

Based on the current evidence, the routine use of tranexamic acid after SAH cannot be recommended. For an SAH patient, it is essential to obliterate the aneurysm as early as possible. In the future, additional higher quality studies are needed to further assess the effect of tranexamic acid on patients with subarachnoid hemorrhage.

## Data Availability Statement

The original contributions presented in the study are included in the article/Supplementary Material, further inquiries can be directed to the corresponding author.

## Author Contributions

MS took responsibility for the integrity of the data and the accuracy of the data analysis. MS and CY drafted the manuscript and performed statistical analysis. MS, Z-hC, and L-fX made critical revision of the manuscript for important intellectual content. W-yZ: supervision. All authors: concept, design, analysis, and interpretation of data.

## Funding

This study was funded by the Key research and development plan of Hubei science and technology department (No. 2020BCB033).

## Conflict of Interest

The authors declare that the research was conducted in the absence of any commercial or financial relationships that could be construed as a potential conflict of interest.

## Publisher's Note

All claims expressed in this article are solely those of the authors and do not necessarily represent those of their affiliated organizations, or those of the publisher, the editors and the reviewers. Any product that may be evaluated in this article, or claim that may be made by its manufacturer, is not guaranteed or endorsed by the publisher.

## Supplementary Material

Supplementary File 1 The PRISMA checklist.

Supplementary File 2 Free terms.

Supplementary File 3 Subgroup analysis of mortality based on treatment duration.

Supplementary File 4 Subgroup analysis of mortality based on follow-up period.

Supplementary File 5A Forest plot comparing tranexamic acid and control treatment for the outcome of adverse events–cerebral ischemia.

Supplementary File 5B Forest plot comparing tranexamic acid and control treatment for the outcome of adverse events–hydrocephalus.

Supplementary File 5C Forest plot comparing tranexamic acid and control treatment for the outcome of adverse events–DVT.

Supplementary File 6A Risk of bias graph: review authors' judgements about each risk of bias item presented as percentages across all included studies.

Supplementary File 6B Risk of bias summary: review authors' judgements about each risk of bias item for each included study.
