# Supplementary material for: Efficacy and Safety of Tranexamic Acid in Aneurysmal Subarachnoid Hemorrhage: A Systematic Review and Meta-Analysis of Randomized Controlled Trials
Source: Front Surg. 2022 Jan 10;8:790149. doi: 10.3389/fsurg.2021.790149 (PMC8784421; doi:10.3389/fsurg.2021.790149)
Supplement: Supplementary File 2 — Free terms. [file Data_Sheet_2.PDF]

Search strategy of PubMed:

(1) ((((((((((((((((((((((((((("Subarachnoid Hemorrhage"[Mesh]) OR (SAH (Subarachnoid Hemorrhage))) OR (Hemorrhage, Subarachnoid)) OR (Subarachnoid Hemorrhages)) OR (Subarachnoid Hemorrhage, Aneurysmal)) OR (Aneurysmal Subarachnoid Hemorrhage)) OR (Aneurysmal Subarachnoid Hemorrhages)) OR (Hemorrhage, Aneurysmal Subarachnoid)) OR (Hemorrhages, Aneurysmal Subarachnoid)) OR (Subarachnoid Hemorrhages, Aneurysmal)) OR (Subarachnoid Hemorrhage, Spontaneous)) OR (Hemorrhage, Spontaneous Subarachnoid)) OR (Hemorrhages, Spontaneous Subarachnoid)) OR (Spontaneous Subarachnoid Hemorrhage)) OR (Spontaneous Subarachnoid Hemorrhages)) OR (Subarachnoid Hemorrhages, Spontaneous)) OR (Perinatal Subarachnoid Hemorrhage)) OR (Hemorrhage, Perinatal Subarachnoid)) OR (Hemorrhages, Perinatal Subarachnoid)) OR (Perinatal Subarachnoid Hemorrhages)) OR (Subarachnoid Hemorrhage, Perinatal)) OR (Subarachnoid Hemorrhages, Perinatal)) OR (Subarachnoid Hemorrhage, Intracranial)) OR (Hemorrhage, Intracranial Subarachnoid)) OR (Hemorrhages, Intracranial Subarachnoid)) OR (Intracranial Subarachnoid Hemorrhage)) OR (Intracranial Subarachnoid Hemorrhages)) OR (Subarachnoid Hemorrhages, Intracranial)

(2) ((((((((((((("Tranexamic Acid"[Mesh]) OR (TXA)) OR (AMCA)) OR (AMCHA)) OR (trans-4-(Aminomethyl)cyclohexanecarboxylic Acid)) OR (t-AMCHA)) OR (Anvitoff)) OR (Cyklokapron)) OR (Ugurol)) OR (KABI 2161)) OR (Spotof)) OR (Transamin)) OR (Amchafibrin)) OR (Exacyl)
