# Supplementary figures and images for: Efficacy and Safety of Tranexamic Acid in Aneurysmal Subarachnoid Hemorrhage: A Systematic Review and Meta-Analysis of Randomized Controlled Trials
Source: Front Surg. 2022 Jan 10;8:790149. doi: 10.3389/fsurg.2021.790149 (PMC8784421; doi:10.3389/fsurg.2021.790149)

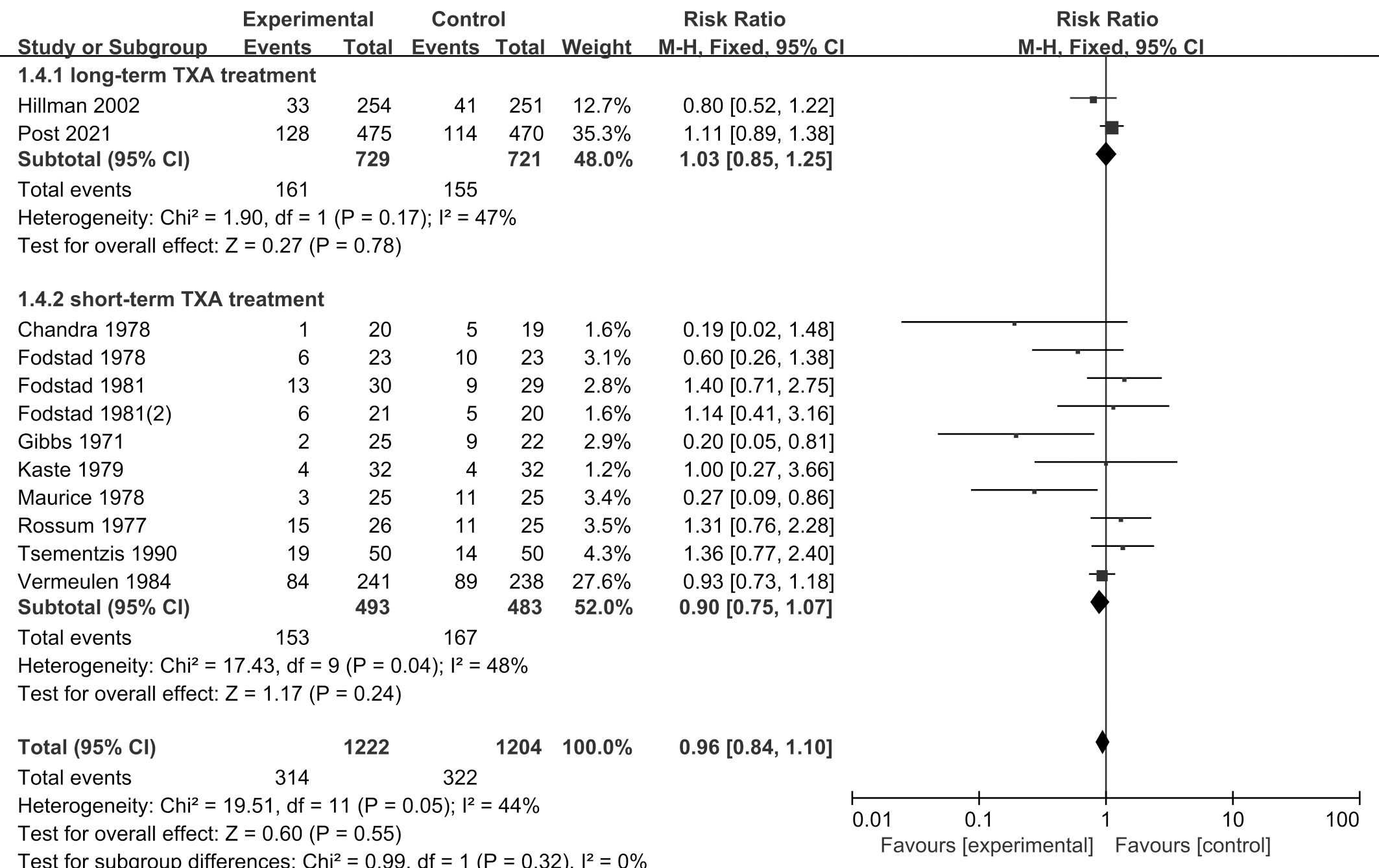

Supplement: Supplementary File 3 — Subgroup analysis of mortality based on treatment duration. [file Image_1.TIF]

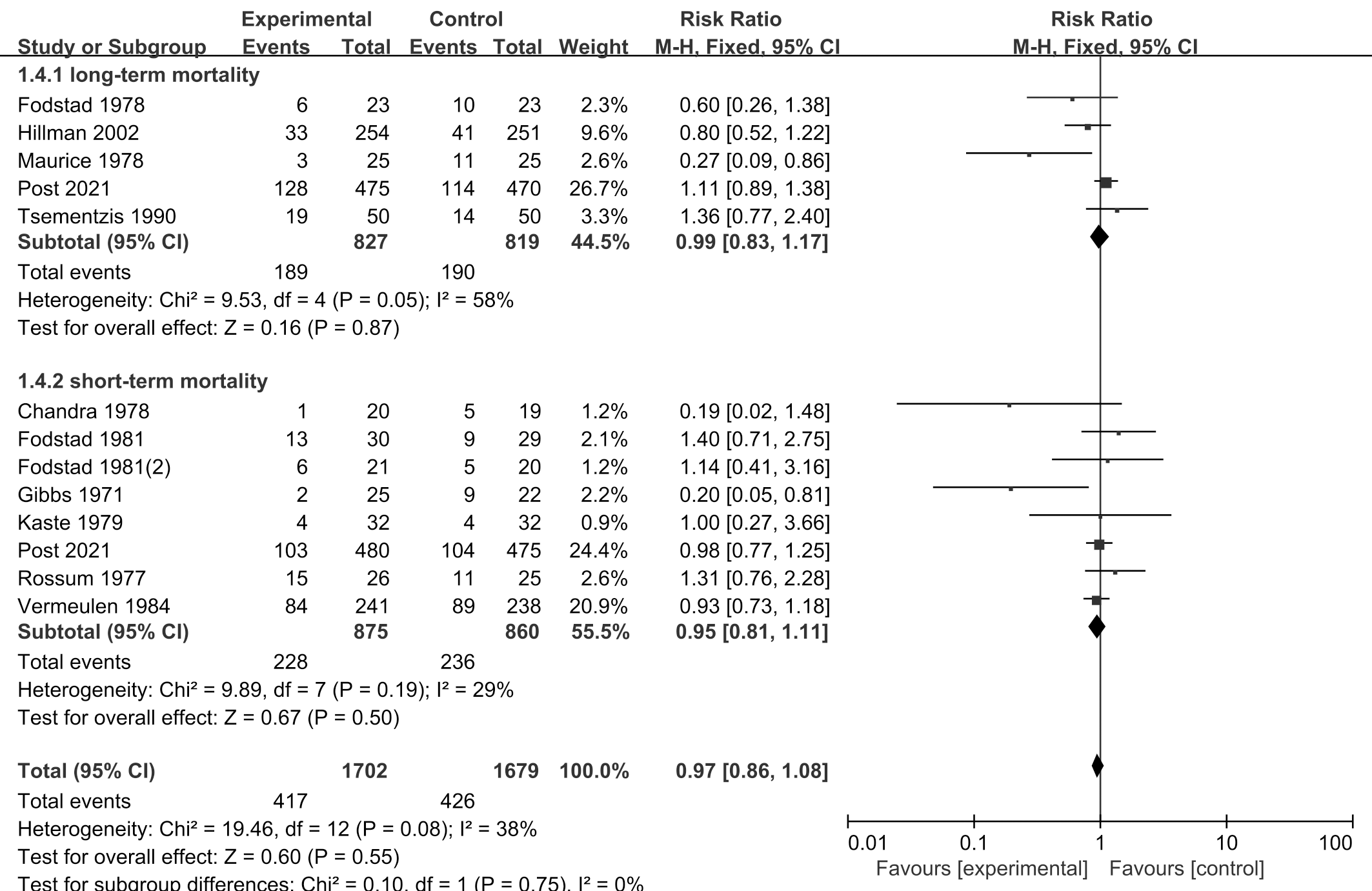

Supplement: Supplementary File 4 — Subgroup analysis of mortality based on follow-up period. [file Image_2.TIF]

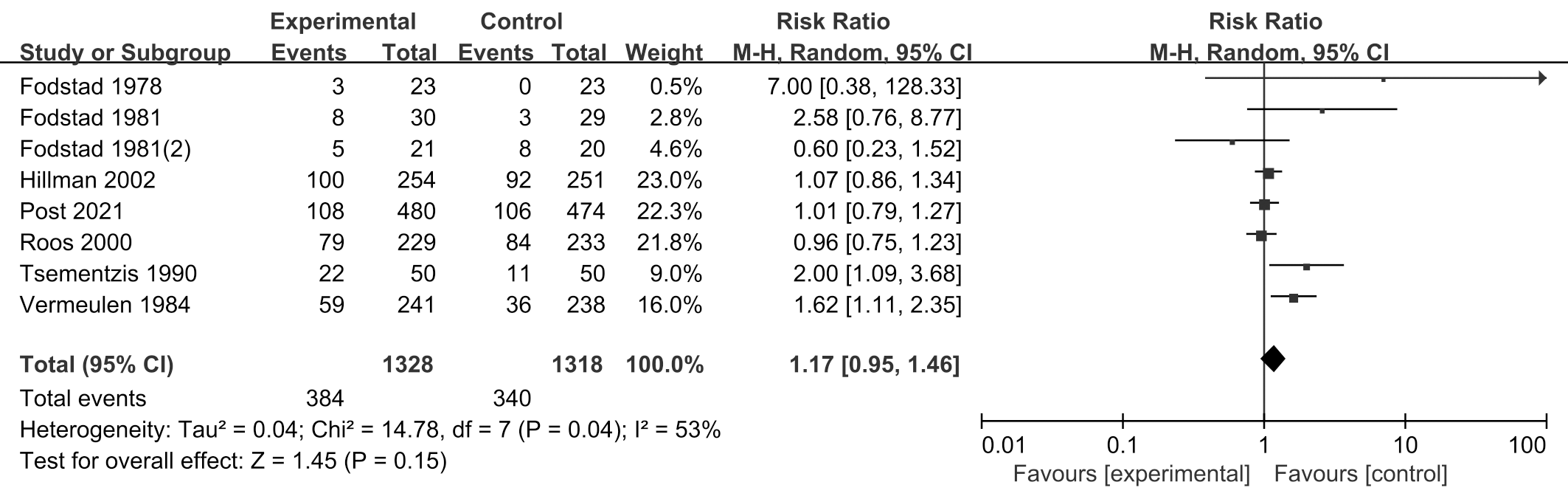

Supplement: Supplementary File 5A — Forest plot comparing tranexamic acid and control treatment for the outcome of adverse events–cerebral ischemia. [file Image_3.TIF]

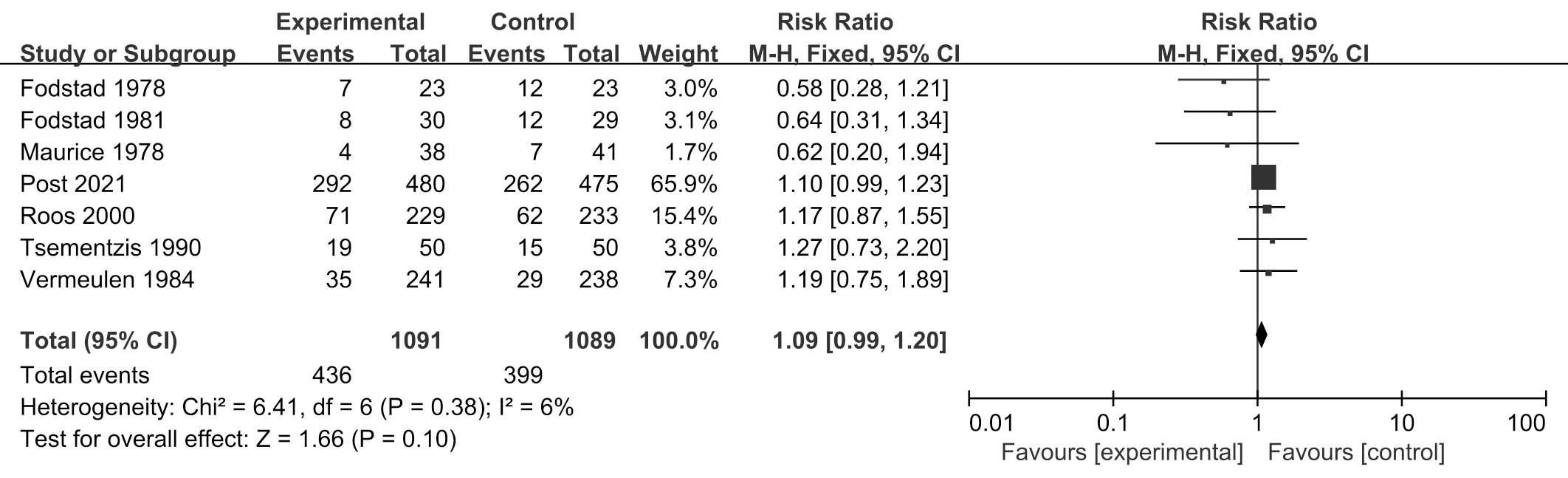

Supplement: Supplementary File 5B — Forest plot comparing tranexamic acid and control treatment for the outcome of adverse events–hydrocephalus. [file Image_4.TIF]

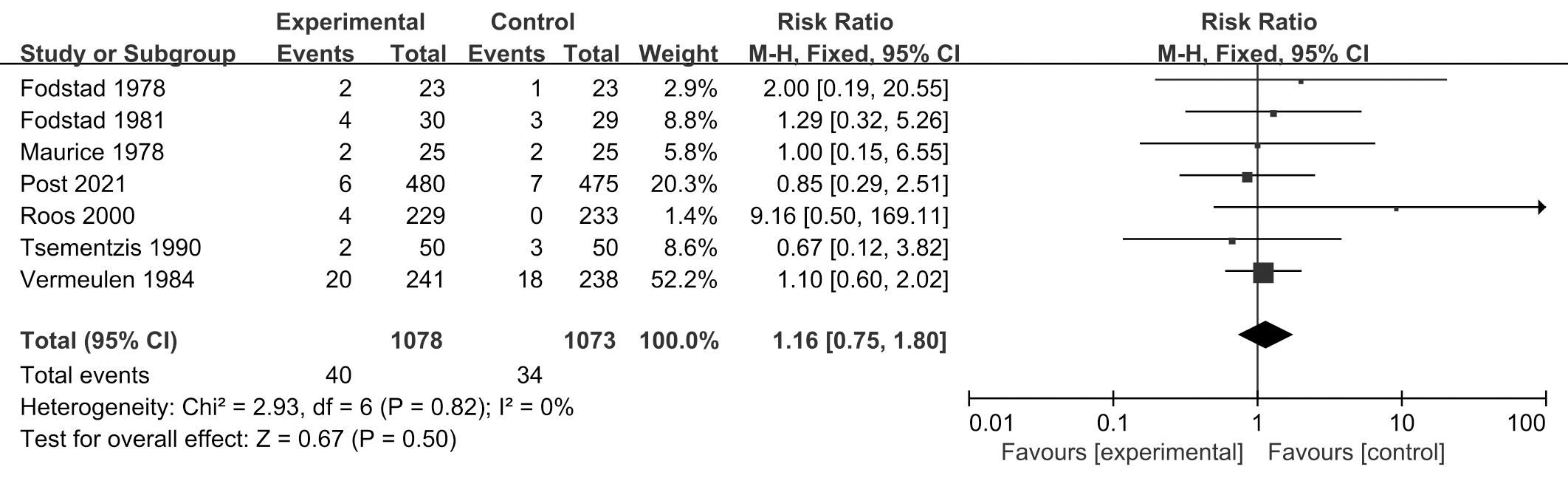

Supplement: Supplementary File 5C — Forest plot comparing tranexamic acid and control treatment for the outcome of adverse events–DVT. [file Image_5.TIF]

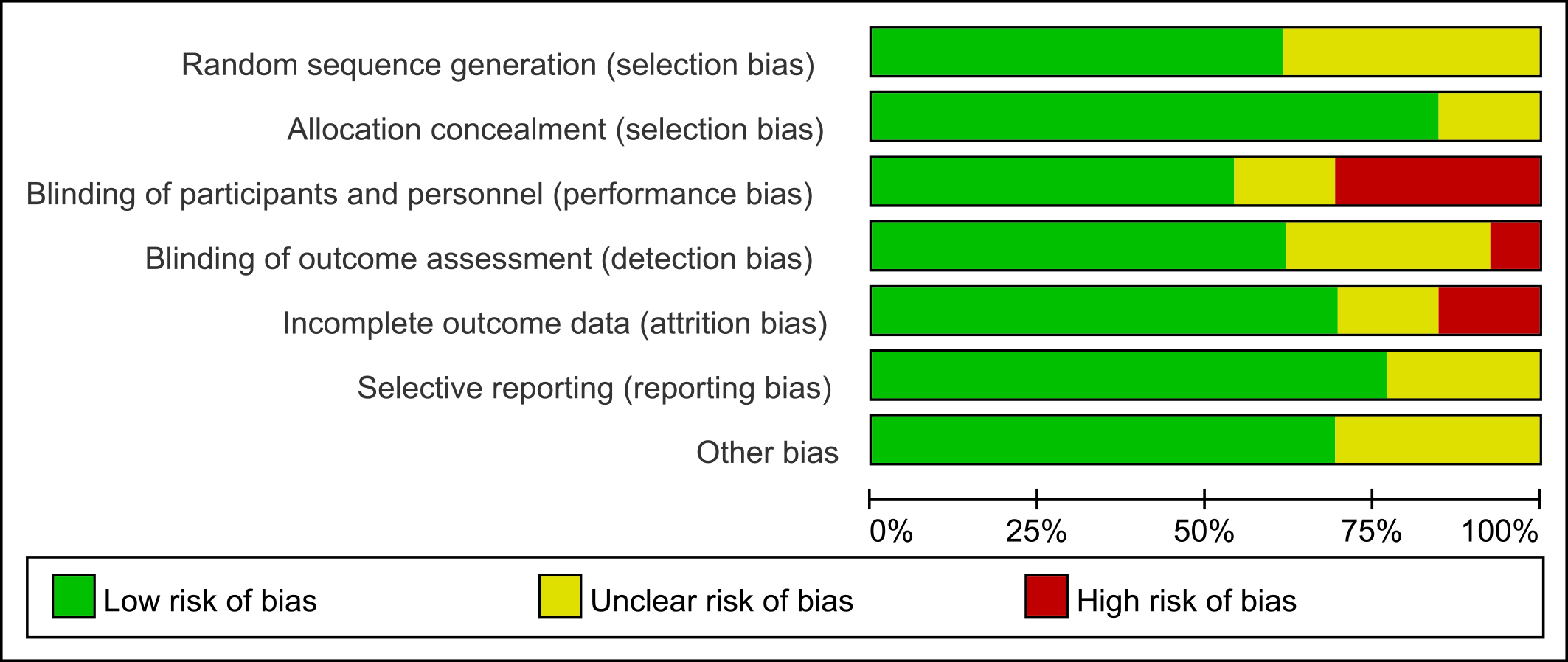

Supplement: Supplementary File 6A — Risk of bias graph: review authors' judgements about each risk of bias item presented as percentages across all included studies. [file Image_6.TIF]

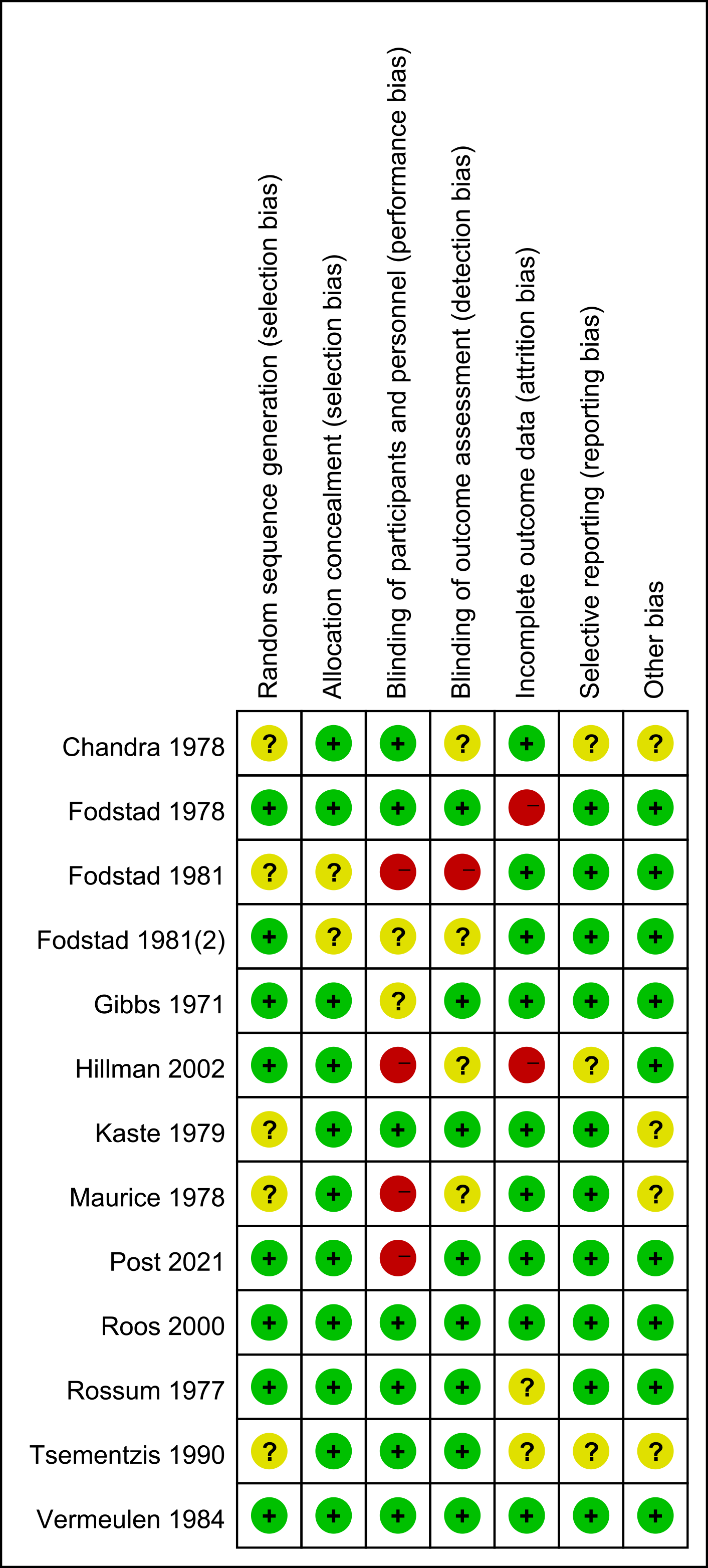

Supplement: Supplementary File 6B — Risk of bias summary: review authors' judgements about each risk of bias item for each included study. [file Image_7.TIF]
